# Consequences of Missed Opportunities for HIV Testing during Pregnancy and Delayed Diagnosis for Mexican Women, Children and Male Partners

**DOI:** 10.1371/journal.pone.0109912

**Published:** 2014-11-05

**Authors:** Tamil Kendall

**Affiliations:** Women and Health Initiative and Takemi Program in International Health, Department of Global Health and Population, Harvard School of Public Health, Boston, Massachusetts, United States of America; UCL Institute of Child Health, University College London, United Kingdom

## Abstract

**Introduction:**

HIV testing during pregnancy permits prevention of vertical (mother-to-child) transmission and provides an opportunity for women living with HIV to access treatment for their own health. In 2001, Mexico’s National HIV Action Plan committed to universal offer of HIV testing to pregnant women, but in 2011, only 45.6% of women who attended antenatal care (ANC) were tested for HIV. The study objective was to document the consequences of missed opportunities for HIV testing and counseling during pregnancy and late HIV diagnosis for Mexican women living with HIV and their families.

**Methods:**

Semi-structured-interviews with 55 women living with HIV who had had a pregnancy since 2001 were completed between 2009 and 2011. Interviews were analyzed thematically using *a priori* and inductive codes.

**Results:**

Consistent with national statistics, less than half of the women living with HIV (42%) were offered HIV testing and counseling during ANC. When not diagnosed during ANC, women had multiple contacts with the health-care system due to their own and other family members’ AIDS-related complications before being diagnosed. Missed opportunities for HIV testing and counseling during antenatal care and health-care providers failure to recognize AIDS-related complications resulted in pediatric HIV infections, AIDS-related deaths of children and male partners, and HIV disease progression among women and other family members. In contrast, HIV diagnosis permitted timely access to interventions to prevent vertical HIV transmission and long-term care and treatment for women living with HIV.

**Conclusions:**

Omissions of the offer of HIV testing and counseling in ANC and health-care providers’ failure to recognize AIDS-related complications had negative health, economic and emotional consequences. Scaling-up provider-initiated HIV testing and counseling within and beyond antenatal care and pre-service and in-service trainings on HIV and AIDS for health-care providers can hasten timely HIV diagnosis and contribute to improved individual and public health in Mexico.

## Introduction

Prevention of vertical (mother-to-child) transmission of HIV is recognized as an important action to achieve the Millennium Development Goals, particularly the health-related goals of reducing child and maternal mortality and turning the tide of the HIV epidemic [1], [2]. Antenatal HIV testing is necessary to prevent vertical HIV transmission and also offers an opportunity for women and other family members to learn their HIV status and access treatment for their own health [3]. In 2012 only 38% of pregnant women in low and middle-income countries were tested for HIV; in the Americas the proportion was 62% [4]. This is despite the fact that in 2001, member countries of the United Nations committed to providing antiretroviral prophylaxis to prevent vertical HIV transmission to 80% of women who needed it by 2011 [5].

After endorsing the United Nations Political Declaration on HIV and AIDS in 2001, Mexico, like many other countries around the world, strengthened domestic policies to prevent vertical HIV transmission. The 2001 Mexican National Action Plan on HIV and AIDS established the universal offer of HIV testing to pregnant women; the 2007–2012 National Action Plan on HIV and AIDS reaffirmed this objective [6], [7]. The most important operational intervention to promote HIV testing during antenatal care (ANC) after 2001 was the national distribution of 800,000 rapid HIV tests to primary care clinics that serve the population without employer-provided health insurance at the end of 2006 and beginning of 2007 with the instruction to offer HIV testing to all pregnant women [8]. However, Mexican HIV legislation was not modified to include the obligation of health-care services to offer HIV testing to all pregnant women until 2010 [9] and, as of June 2014, the national ANC legislation still stated that antenatal HIV testing should only be offered to “high risk women–those who have received blood transfusions, drug addicts, and prostitutes” [10]. This disjuncture between the commitment to offer of HIV testing to all pregnant women in the HIV National Action Plan and the legislative frameworks for HIV and reproductive health care, combined with the vertical organization of HIV and reproductive health services, assignation of the responsibility for prevention of vertical HIV transmission to the HIV program, and competing priorities in both HIV and reproductive health have been identified as policy and political barriers to the scale-up of HIV testing among pregnant women in Mexico, as well as other Latin American countries [11], [12]. Additional operational barriers to antenatal HIV testing identified in Mexico between 2001 and 2011 included a limited number of health workers trained in HIV testing and counseling in reproductive health services and insufficient availability of commodities [12].

Mexico’s national prenatal care legislation has recommended a minimum of five ANC visits since 1993 and the national HIV action plan introduced universal offer of HIV testing in 2001 [6], [10]. Since 2006, 81.5% of women have begun ANC during the first trimester and beginning in 2009 national guidelines for antiretroviral management recommended HIV testing during the first trimester of pregnancy or as soon as possible thereafter [13], [14]. However, when the current study began in 2009, despite 95.8% of Mexican women attending at least one ANC visit and 86.3% of women attending four or more ANC visits [15], only 42.1% of Mexican women who attended ANC were tested for HIV [16]. In 2011 when the study concluded, only 45.6% of women who attended ANC were tested for HIV [16]. The objective of this analysis is to describe missed opportunities for HIV diagnosis during pregnancy and document the consequences for the health and well-being of women living with HIV and their families.

## Methods

### Ethics statement

The design and conduct of this study was reviewed and approved annually by the University of British Columbia-Okanagan Behavioural Research Ethics Board and the Mexican National AIDS Program (CENSIDA) Ethics Board. To ensure confidentiality, pseudonyms were assigned to all women living with HIV who participated in the research, and physicians and decision-makers are identified only by their professional role.

### Data collection and analysis

The analysis draws on semi-structured interviews with 55 Mexican women of reproductive age living with HIV conducted between July 2009 and January 2011. The criteria for women’s inclusion in the study were: living with HIV, speaking Spanish, being at least 18 and of reproductive age (18–49 years of age), and having had a pregnancy since 2001 when universal offer of HIV testing during ANC was first included in the Mexican National Action Plan on HIV and AIDS. Women living with HIV were recruited from eight ambulatory HIV clinics in Central Mexico (Mexico State, Morelos, and Mexico City) that serve the population without employer-provided health insurance. The population attending these health services is generally economically disadvantaged. Reported monthly family incomes of the women living with HIV ranged from 200 pesos (USD 16) to 10,000 pesos (USD 777) per month, with a median family income of 3000 pesos per month (USD 233) and a mean income of 3500 pesos per month (USD 272). All of the women living with HIV reported monthly household incomes below the Mexican national mean of USD 950 a month [17]. The exchange rate of 12.86 Mexican pesos for a US Dollar was calculated using the Federal Reserve Rate on the first day of each month between July 2009 and December 2010 (http://www.federalreserve.gov). Based on the cost of the basic monthly food basket in urban areas in 2008, which Mexico uses to estimate poverty, two-thirds of the women faced food insufficiency at the household level even before taking into account the cost of rent and other living expenses [18].

Women of reproductive age living with HIV were given a written invitation to participate by clinic staff. If they met the selection criteria and wished to participate, clinic staff made an appointment to conduct an interview. Interviews were conducted in Spanish by the author, who has been doing qualitative research with Mexican women living with HIV for more than a decade, or a female Mexican anthropologist experienced in reproductive health research who was trained by the author about HIV and in the use of the interview guide. A site which afforded sufficient privacy was chosen by the woman living with HIV; most interviews were conducted in a private office at the HIV clinic. Research participants were given 100 pesos (approximately eight dollars) to pay for their travel and childcare. All research participants signed written informed consent prior to the interview. As one of the study objectives was to analyze implementation of the Mexican program to prevent vertical HIV transmission, and consequences of successful or failed implementation for women and their families, this was a purposive sample of women living with HIV diagnosed during ANC as well as women who were diagnosed in other settings. Sample size was determined by the principle of saturation, which refers to the point at which additional interviews do not provide novel information about the primary areas of interest [19]. Interviews were continued until saturation was reached with respect to women’s narratives about the offer of HIV testing during antenatal care, the circumstances of their HIV diagnosis, and referral to services to prevent vertical HIV transmission. Numerical balance between women living with HIV diagnosed during ANC and those diagnosed in other settings was not sought.

Interviews with women living with HIV ranged from one to two hours in length and included questions about the circumstances of the HIV diagnosis, pregnancy history, sexual and reproductive practices and desires before and after the HIV diagnosis, experience of and management of HIV disease, prevention of vertical HIV transmission, interactions with the health-care system and health-care providers, and social and economic situation. This analysis focuses on the experiences of women living with HIV however data from 60 additional interviews with health-care providers, policymakers and other experts about prevention of vertical HIV transmission in Mexico between 2001 and 2011 are drawn upon to contextualize women’s experiences.

All interviews were audiotaped and transcribed verbatim. The author conducted analysis during fieldwork and modified the interview guide to explore emerging issues. Interviews were coded thematically by the author, using a combination of predefined codes related to areas of interest and codes which were generated inductively from the interviews. Examples of *a priori codes* were HIV diagnosis during ANC and circumstances of the HIV diagnosis; examples of inductive codes include diagnosis because of AIDS-related morbidity or mortality of a child or a male partner, diagnosis when women developed AIDS-related complications, or through provider-initiative testing and counseling outside of ANC. Data were managed using the qualitative analysis package Atlas-ti 6.0.

## Results

**Figure 1:**
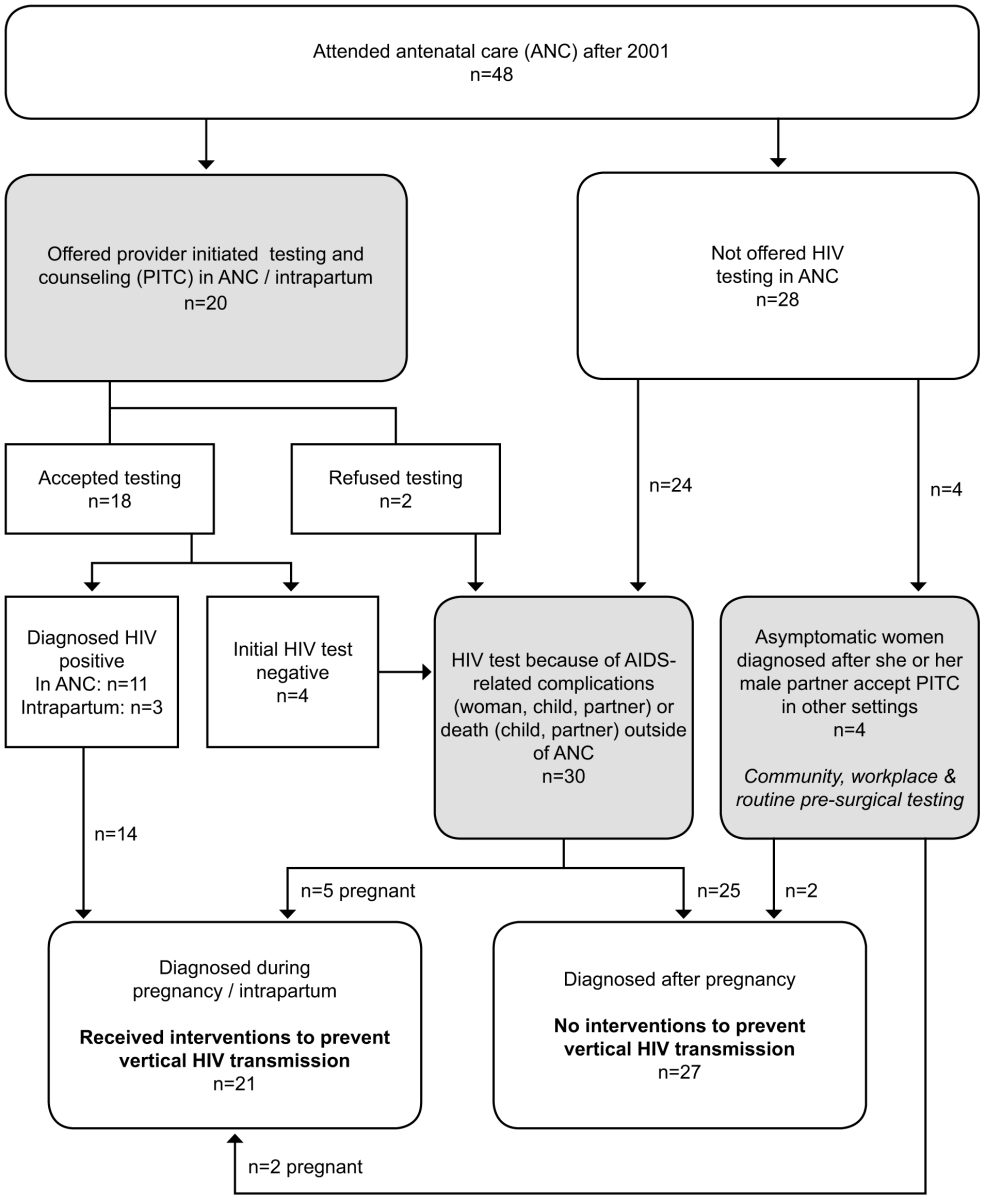
Routes to HIV diagnosis for Mexican women who attended antenatal care (ANC) after 2001.

In total, 48 of the 55 women living with HIV attended ANC without knowledge of their diagnosis between 2001 and 2009. Figure 1 describes two routes to HIV diagnosis for these 48 women who attended ANC after 2001 and were diagnosed subsequently with HIV: 1) provider initiated testing and counseling (PITC) as part of ANC or intrapartum care, or 2) missed opportunities for HIV diagnosis during ANC/intrapartum care and subsequent HIV testing in another setting, usually when the woman, her child(ren), or the male partner developed AIDS-related complications.

As shown in Figure 1, all 14 pregnant women who were offered PITC and tested positive during ANC or intrapartum care received interventions to prevent vertical HIV transmission, as did another seven women who were diagnosed with HIV during pregnancy outside of ANC (21/48). Of the 34 women who were not diagnosed in ANC/intrapartum care, 27 were diagnosed after delivery and did not receive any interventions to prevent vertical HIV transmission (27/48). Four women who were offered and accepted PITC during ANC or intrapartum care had an initial negative test result. These four women, two women who refused HIV testing during ANC, and twenty-four women not offered PITC in ANC were subsequently diagnosed when the woman, her child(ren), or the male partner became ill with AIDS-related complications (30/48).

Seven women living with HIV were excluded from Figure 1 because they were already aware of their HIV diagnosis before becoming pregnant and accessing ANC post-2001. Their routes to HIV diagnosis were similar to other women who were not diagnosed during ANC: the woman becoming ill with AIDS-related complications, the male partner becoming ill or dying of AIDS-related complications, or PITC of asymptomatic women in other settings e.g. at work or prior to surgery.

### HIV diagnosis during pregnancy

On average, women went to 6.5 ANC visits in the pregnancy prior to HIV-diagnosis or the pregnancy during which they were diagnosed [range 1–10], providing ample opportunities for health workers to offer HIV testing. Nevertheless, of the 48 women who attended ANC after 2001 but before being diagnosed with HIV, only 20 (42%) were offered HIV testing during ANC or labor and delivery (Figure 1). Many of the women who reported antenatal HIV testing were pregnant during 2007 and 2008 (after the national distribution of rapid tests to public primary care clinics), however other women who attended ANC in those years were not offered testing. The haphazard offer of HIV testing and counseling during ANC is exemplified by Nancy’s experience. In 2008, she attended ANC at the public primary health care center “in my neighborhood, where my mother lives” and was not offered HIV testing and counseling. Nancy only learned she was living with HIV during pregnancy because, by chance, she went to an ANC visit at a different public primary health care center in the same city and was offered an HIV test.

Of the 20 women who received PITC during ANC, two refused testing, for an acceptance rate similar to the 85–90% identified in other Mexican studies [20], [21]. Women who refused testing said they had not received specific information about preventing vertical HIV transmission as part of counseling. The perception among a few women that HIV testing was compulsory during pregnancy also suggests that the quality of counseling was sub-optimal. For instance, Jacinta wanted to be tested but understood HIV testing to be “a requirement of the health center to receive prenatal care”.

Four of the women tested during ANC received negative test results (Figure 1). While one woman reported that she acquired HIV from a partner who she met after the index pregnancy, the other three women had the same male partner at the time of diagnosis as during the index pregnancy and were diagnosed shortly after delivery. This finding indicates that repeat HIV testing in the third trimester of pregnancy could provide additional benefit for identifying women living with HIV, preventing vertical HIV transmission, and enabling women’s timely referral to long-term care.

Another group of women (n = 7) were not offered HIV testing as a routine part of ANC but were diagnosed during pregnancy. Among these women, four were diagnosed because their male partner became ill with AIDS-related complications during the pregnancy, one male partner disclosed his HIV status after his female partner became pregnant, one woman’s husband received PITC as part of his application for work, and one woman happened to test at a street testing fair held in her neighbourhood.

### Provision of interventions to prevent vertical HIV transmission and linkage to long-term HIV care and treatment

Once women had an HIV-positive test result, health-care providers and administrators reported that they expedited confirmation of pregnant women’s HIV test results and access to interventions to prevent vertical HIV transmission, including antiretroviral prophylaxis. The head of a state HIV Program, explained “we consider a pregnant woman an emergency so we don’t wait to have the viral load and CD4, we start the [prophylactic antiretroviral] regimen immediately.” These efforts were reflected in women’s reported experiences. Of the 21 women who learned their HIV status during pregnancy, all took steps to prevent vertical HIV transmission: 17/21 received antiretroviral prophylaxis; one woman diagnosed at 38 weeks gestation delivered by cesarean section and her child was given antiretroviral prophylaxis; none of the three women diagnosed during labor received antiretroviral prophylaxis for themselves or their children but two delivered by caesarean section; and none of the women breastfed. Of the 21 women who learned their diagnosis during ANC or intrapartum, 16 had children with an HIV-negative diagnosis, three children were too young to have a confirmed diagnosis, and two women were pregnant at the time of the interview.

With respect to linkage to and uptake of long-term HIV care, whether they were diagnosed during prenatal care or in another context, most women reported a relatively smooth and speedy transition from initial diagnosis to being referred to a public HIV clinic, confirmatory testing of HIV status and receiving the first CD4 and viral load count. At the time of the interview, all of the participants were enrolled in long-term HIV care and treatment for their own health. Sara’s experience was typical of that reported by most participants, in that during the month after her child was born she “started to go to the [HIV] clinic with the doctor, my medical visits, my medications, and did everything to take care of myself.” However, 7 of 55 women (12.7%) reported delaying seeking care and treatment after their diagnosis (from a few months up to several years), suspending HIV care for a period before they were treatment eligible, and temporarily stopping treatment during the postpartum period; only one of these women was diagnosed during pregnancy. Reasons given by women for delaying or suspending HIV care and treatment included lack of knowledge about HIV disease and treatment, perceived lack of empathy from health-care providers, antiretroviral side effects, prioritizing a child’s treatment over their own health, and the cost of transportation. For example, Amparo said that before she was eligible for treatment she “stopped coming for about a year. I thought ‘I’m fine, the doctor doesn’t even give me a check-up, so why I am paying for transport and leaving my little kid.”

### Health, economic and emotional consequences of missed opportunities for HIV testing and delayed diagnosis

When the opportunity to offer HIV testing during ANC was missed, the most common route to the HIV diagnosis was when women or their family members experienced AIDS-related complications (Figure 1). As discussed in more detail below, the health, economic and emotional consequences of missing the opportunity to diagnose HIV during pregnancy were frequently exacerbated by health-care providers’ failure to recognize HIV and AIDS-related complications among children, male partners, and women.

### AIDS-related morbidity and mortality among children

In this sample, only women who were not diagnosed with HIV during pregnancy had children who were known to be living with HIV, or who had died from complications that were known or suspected to be AIDS-related. Four of the women interviewed experienced the death of a child who tested positive for HIV, three lost a child who had symptoms that could be due to AIDS without a diagnosis, and ten were mothers of children living with HIV. Children’s AIDS-related illness or death was a common way for women to learn their HIV diagnosis. The tragedy of the preventable child death due to vertical HIV transmission is compounded by the fact that the parents invariably sought medical care for their children without receiving a timely HIV diagnosis. Itzel and her husband were typical in this respect. Despite their limited income (she is a maid and he is a day labourer), they sought specialized medical care in the public and private sectors for their son, and became deeply indebted. Itzel said:

Test after test, they pricked him wherever they wanted and they never did this one [HIV], until the very last days when he had a convulsion and then, according to them, they wanted to do a more advanced test. By then we had gone through everything. Paediatricians and paediatricians, private ones, and the best–we spent so much money. […] And we got deeply into debt. And for what? Nothing. Doctors and doctors and nothing. Why did it not occur to them to think just for a minute about [HIV]? And we did not either because, well, we never could have imagined it.

By the time of her son’s death from AIDS-related complications at two years of age in 2008, Itzel had also progressed to AIDS. Mother and child had the same clinical symptoms: extreme weight loss (wasting), vomiting, and diarrhoea. Itzel explained that it “seemed strange to me that I had the same symptoms as him, you understand? I had totally lost weight, a lot of diarrhoea, and everything that we ate, I threw up, and that was how he was.” If Itzel’s husband had not taken the initiative to track down the physician who had ordered the HIV test for their son because he wanted to know what his son had died of, even their son’s death would not have resulted in them learning their HIV diagnosis.

Similar to Itzel, Karen sought health-care for her son but did not learn he was living with HIV until shortly before he died. Karen’s son died of AIDS-related complications at three years of age, and her younger daughter is HIV-positive. The history of Karen’s pregnancies and the AIDS-defining illnesses experienced by the family illustrate that health-care providers had multiple opportunities and indications for recommending HIV testing. During her first pregnancy in 2004, Karen and her husband were both public sector employees with health insurance. Yet despite attending eight ANC visits and doing “eight ultrasounds with my son, every month I did one,” Karen was not offered HIV testing and counseling. Her son was born vaginally and she breastfed exclusively for three months after which time she fed him with formula and breast milk. He was a sickly child and was being hospitalized regularly for infections and respiratory problems when she became pregnant with her daughter. During her second pregnancy in 2006 Karen attended six ANC visits but was not offered HIV testing. Her son’s repeated illnesses, her husband’s AIDS-defining illness (oesophageal candidiasis) and both of them being hospitalized did not result in health-care providers recommending HIV testing. Karen said she feels “impotent about my son because we knew that my husband got sick a lot from this. He was hospitalized several times and they never, ever diagnosed anything”. Just as her husband’s symptoms did not provoke suspicion of HIV, her son was also misdiagnosed. Karen was told her son “had adenoiditis, and that was why he was always getting sick in his throat. So we finally got together enough money, and my father also lent us [money], so that he could have an operation.” After the operation, Karen’s son became acutely ill and remained hospitalized. At the same time, his younger sister became ill and was hospitalized. Karen related that “when they saw the two little siblings hospitalized, they asked why. It seemed strange to them, and they started to do tests”. The simultaneous hospitalization of Karen’s two children led to Karen, her husband and their two children learning that they were living with HIV.

Three other women living with HIV had lost children due to symptoms that could be AIDS-related but who died without a diagnosis. For example, Lilia miscarried and then, “in 2005, I had another baby who died at twenty-seven days old. He got pneumonia in one of his little lungs.” Antonina’s daughter died, but “even now, three years later, I don’t know what killed her. She was sick to her throat. We took her to the doctor, but never found out what was wrong with her.”

### AIDS-related morbidity and mortality among male partners

Another route to the HIV diagnosis for women was when a male partner became ill with AIDS-related complications. In Carmen’s case, health-care services missed the opportunity to diagnose her in 2003 while she was pregnant, but when her husband became ill with AIDS-related complications in 2009, they were both diagnosed. Similarly, Luisa was not offered HIV testing and counseling though she attended ANC during three pregnancies between 2002 and 2006. She learned she was HIV-positive in 2008 when her husband “started to get sick with fever, his whole body hurt, his bones, and when we came to the hospital for a vaccine, because he felt sick, the doctor told me that he did not have anemia, that probably he had AIDS.” She and her husband both tested positive for HIV, and he died shortly afterwards. Very late diagnosis and deaths from AIDS-related complications were a hallmark of the family medical histories of women who participated in the research.

### Women’s HIV disease progression

The other common route to diagnosis was when a woman presented symptoms of HIV disease or AIDS-related complications. However, these diagnoses were frequently delayed. Over and over again, women reported that they had sought health care for a variety of HIV-related ailments without physicians ever suggesting an HIV test. Paola’s story exemplifies the difficulty that women have receiving an HIV diagnosis despite being symptomatic. She said that

for almost a year I was doing tests. I was going to urologists and other specialists because it seemed that I had a urinary tract infection. I had pain that wouldn’t go away. I went to several different physicians but none sent me to do the [HIV] test. It was only when I felt really sick, I had a lot of diarrhoea and fever, I went to a doctor who knows my father, a family doctor. And because I trusted him, and based on the symptoms, the medicines [I had taken], and that I hadn’t gotten better, he sent me to do an ELISA [Enzyme-linked immunosorbent assay].

Research has shown a close relationship between gynaecological disorders and HIV among women, yet despite her symptomology and determined health seeking, Paola progressed to the third clinical stage of HIV disease before she received her diagnosis [22]. Noemi had different symptoms than Paola, but she also experienced a long period of health-care seeking and became very ill before she was diagnosed.

They told me that it was pneumonia. They controlled it, I was okay, and then I got sick again. After that, they did tests but they never did one of these [HIV] tests. Because they did not think that it could be this [HIV]. They said that no, well it was just pneumonia, it was pneumonia, and they prescribed medicine, and I got better, and then again.

Noemi presented with recurrent pneumonia, an AIDS-defining illness, for years without an HIV test being suggested. When Noemi was finally tested for HIV, she was extremely ill:

They put me in intensive care because I had third grade dehydration; they said I arrived weighing thirty kilos (66 lbs.). By then I had pneumonia, dehydration, I was in the third stage of HIV. And as well, I had genital herpes, and also in the mouth. I was at the end. In fact, they [the physicians] told my family to come and say goodbye because they couldn’t do anything for me.

Women living with HIV repeatedly narrated becoming very ill–losing weight, having fevers and diarrhoea, a chronic chest infection or pneumonia, or on-going gynaecological complications, and consulting a variety of physicians without receiving the recommendation of an HIV test.

### Economic consequences

Futile health-care seeking that included consultations with specialists who ordered numerous laboratory tests but never recommended HIV testing, prescribed medicines and performed surgical interventions to treat AIDS-related symptoms and disease progression that limited women and men’s ability to work had negative economic consequences for families. In many cases, the families of these women living with HIV simply could not afford their odyssey through the health system, and became indebted in order to pay their medical bills. Itzel explains that when seeking a diagnosis for her now deceased son, “sometimes my husband said, or I said: ‘Well, now there is no money.’ And then he’d say: ‘well, we’ll get it from wherever we can, what’s important is that he’s healthy and well.’” When men and women experienced AIDS-related complications, they became unable to work, digging their families into an ever deeper economic hole. For instance, Pamela was not diagnosed with HIV during her first pregnancy. While she was pregnant with their second child, her husband became ill with AIDS-related complications. The family was forced to migrate from another state to live with her male partner’s family, increasing the economic precariousness of two households. Pamela said her greatest fear was her husband getting

sick again, right now he is in bed, and he cannot do almost anything …. He will not be able to work until I don’t know when. We rely on my father-in-law. And it’s not the same as before. Before, my husband gave me my money, and I could buy what I wanted–things for my daughter. And now it is very different. We are dependent on my father-in-law, and with him it’s only food, and hospital bills.

Death or abandonment by the male partner also left women and children in difficult economic situations. For example, at the time of the interview in March 2010, Anel was the only economic support for her family. She described being the sole breadwinner and the primacy of her job as a burden and a barrier to her HIV treatment: “I arrive too early for my appointments, and I apologize a million times, because at work they don’t give me time off. And if I lose [my job] how do I eat? It is the only thing I have to support my kids.”

### Emotional consequences

The emotional costs of the failure to prevent vertical HIV transmission were also high, both for those women who had children living with HIV and those whose children had died of AIDS-related complications. A psychologist working in an ambulatory HIV clinic called the transmission of HIV to a child a “double grief” for their mothers.

I am speaking about part of the Mexican culture in which the value of maternity is channelled into moral questions, questions of values, of virtues, right? A good woman is a good mother, a good mother gives everything for her child, and a good mother who gives everything for her child would not forgive herself for an irresponsible act or let herself be, let herself be-I don’t know how I can express this–to not provide protection to avoid that the child be born infected. Based on my work experience, this is how I could sum it up: when children are born infected because they did not realize in time, the mothers experience a double grief and they have to work doubly hard to accept [the diagnosis]. First, to accept that they are the ones who are alive. And afterwards to work out the guilt that they were the ones who infected their children.

Women’s guilt because they had, unknowingly, transmitted HIV to their children was a recurrent theme of the interviews. Gisela said that having a child who is living with HIV “isn’t nice, because they are going to suffer. Right now my daughter is little and everything, but when she is bigger, she is going to say that it is my fault.” Karen, who was inconsolable about the AIDS-related death of her son and wept for most of the two hour long interview, said that “it hurts me because he was a child and he wasn’t guilty of anything. I feel that I am [guilty] because I did not take care of myself, and I never realized that he was sick [with AIDS-related complications].” Karen never suspected she could be living with HIV, assiduously sought health-care services during pregnancy and to promote her son’s health, and still blames herself for his death. The cultural construction of motherhood that condemns women for their children’s ill health, irrespective of women’s circumstances and efforts to promote their children’s well-being, is made clear by Lourdes’ experiences with health-care providers when seeking care for her son before either of them were diagnosed with HIV. Lourdes went to the regional hospital with

a bag of [his] medicines and prescriptions and I don’t know what all. And even then the doctors who were there hit me with everything they had. They said, ‘What an irresponsible mother. How is it possible that she is just letting this child die? Can you believe it?’ ‘No Miss, [Lourdes responded], I am not letting him die. Here are all of his prescriptions, all of his medicines, everything that I am giving him, and he just doesn’t get better.’ ‘Why?’ I also wanted to know what was wrong with my child.

Vertical HIV transmission, disability and death among children is mainly attributable to health-care providers’ failure to offer HIV testing and counseling during pregnancy and failure to recognize AIDS-related complications among children, yet women living with HIV experienced guilt and were blamed by health-care providers for their children’s ill-health.

## Discussion

One of the central findings of this study is that these women were in regular contact with the health-care system for ANC and on multiple other occasions while seeking a diagnosis for AIDS-related complications for themselves or other family members and that often HIV counseling and testing was not offered. For more than half of the women living with HIV, the first failure to offer HIV testing and counselling occurred when they attended ANC during pregnancy. This omission of the offer of HIV testing and counselling was then frequently repeated when women and their families subsequently sought health-care for HIV and AIDS-related complications. Consequences of the omission of the offer of HIV testing and counseling during ANC and then during repeated contacts with the health care system included: new pediatric HIV infections, including younger siblings becoming infected with HIV because the woman wasn’t diagnosed during the earlier pregnancy, disease progression of women, children and male partners, and deaths due to AIDS-related causes among children and male partners. The women living with HIV who participated in the research had survived these omissions. Beyond negative health consequences, futile health-care seeking and delayed diagnoses had negative economic and emotional consequences for women living with HIV and their families.

The serious health, economic and emotional consequences of the failure to offer HIV testing and counseling during ANC and delayed HIV diagnoses for women and their families could be averted by: 1) routine PITC in ANC and 2) improved recognition of AIDS-related complications by health-care providers.

To address low levels of HIV testing during ANC internationally, both the Centers for Disease Control and Prevention in the United States and the World Health Organization have advocated routine PITC [23], [24]. Studies in both high- and low-income countries have demonstrated that antenatal PITC results in significantly higher HIV screening rates and increased implementation of programs to prevent vertical HIV transmission without a corresponding drop in clinic attendance because of fears of HIV testing [25]–[28]. The high uptake of PITC described in this study reiterates other Mexican studies that have found that PITC in ANC is acceptable to a large proportion of pregnant women with HIV (85–90%) and emphasizes the importance of counseling that informs pregnant women that vertical HIV transmission exists, that it can be prevented, and that there is effective and free treatment for HIV disease available for women’s own health [20], [21]. The need to improve and monitor the quality of counseling is also highlighted by the finding that a few women understood testing during pregnancy to be compulsory, substantiating concerns that PITC can contravene principles of informed consent; it is crucial to monitor PITC to ensure informed consent and confidentiality [24], [29], [30].

This research also suggests that the failure to offer HIV testing and counseling in Mexico is not simply an issue of financial constraints-either for the health-care system or for individual families. Frequently ANC included many tests and procedures which are more expensive than an HIV test. In fact, some of the care provided could be considered superfluous. The most dramatic example of this was reported by Karen who underwent eight ultrasounds during ANC but was not offered an HIV test–this omission resulted in the death of her son from AIDS, the subsequent transmission of HIV to her younger daughter, and delayed her and her husband’s HIV diagnosis until he was experiencing AIDS-related complications. In response to AIDS-related complications, these low-income families actively sought health-care in both the for-profit and not-for-profit sectors, and paid for physician visits, laboratory tests, medications, and surgery.

Late HIV diagnosis is a serious individual and public health problem in Mexico and around the globe. For individuals living with HIV, late diagnosis (lower CD4 count, higher viral load, or an AIDS-defining illness) has been associated with a greater probability of progression to AIDS and death [31]. In Mexico, half of those people newly diagnosed with HIV in 2011 were categorized as having AIDS [32]. Similarly, a study from a tertiary level Mexico City hospital categorized 61% of people as late-testers because within six months of diagnosis, they had CD4 counts below 200 and/or they had experienced an AIDS-defining illness [27]. Free lifelong antiretroviral treatment for people living with HIV has been available to beneficiaries of the Social Security Institutes for private and public sector workers since the late 1990 s and those without employer-provided health insurance since 2003 [33]. However, many of the women living with HIV who participated in this study and their family members weren’t able to take advantage of freely available antiretroviral treatment in a timely fashion because of delayed HIV diagnosis. This study makes a novel contribution to the literature by documenting how missed opportunities to offer HIV testing and counseling to women when they attend ANC during pregnancy and to recognize the symptoms of HIV and AIDS when women and other family members seek health-care resulted in late diagnosis.

After the HIV diagnosis, women’s experiences of linkage to and uptake of health-care services suggest both the existence of a relatively strong referral system within the public health-care system and women’s strong motivation to prevent vertical HIV transmission and access long-term HIV care and treatment for themselves and other family members. At the time of the interview, all of the women living with HIV were participating in long-term HIV care and treatment. A small proportion of women (7 of 55, 12.7%) reported that they had delayed or interrupted HIV care and treatment for their own health. Reasons given, such as lack of knowledge about HIV disease and treatment, not having symptoms, negative interactions with health-care providers, and transportation costs have also been identified in other settings and should be addressed [34], [35]. Research from sub-Saharan Africa has raised concerns about the large proportion of women (ranging from 38% to 88% depending on the study) who do not enroll in care and treatment for their own health after being diagnosed with HIV during pregnancy [35], [36].

### Study limitations

The experiences of women attending public HIV clinics located in three Central Mexican States may not be relevant for women of different socioeconomic status, or those who live in other areas of the country. Despite increasing coverage of HIV testing during the period under study, omission of HIV testing in ANC remains a significant problem in Mexico. The most recently published national statistics indicate that in 2012 only 59.8% of pregnant women who attended ANC were tested for HIV [16]. In addition, as women were reporting on events that could have occurred up to a decade prior to the interview, recall bias may have influenced their responses. Finally, as this study recruited women living with HIV from care and treatment clinics, it cannot provide insight into the linkage to and uptake of long-term HIV care and treatment by all women testing for HIV during ANC. Studies to explore this issue should be considered a priority for HIV research in Mexico and other Latin American countries.

## Conclusions

A decade after universal HIV testing for pregnant women was included in Mexico’s National Action Plan on HIV and AIDS, less than half of Mexican women attending ANC were tested for HIV [6], [16]. The seriousness of the negative health, economic and emotional consequences of this omission for women and their families and the high acceptance rate of HIV testing by pregnant Mexican women are compelling arguments firstly to create a favorable policy environment by harmonizing ANC legislation with existing HIV legislation and secondly to make the necessary investments in health-care provider training, commodities, supervision and monitoring to rapidly accelerate scale-up of routine provider-initiated HIV testing and counseling during ANC. This research also documents the need to undertake national education campaigns for health-care workers about the HIV epidemic and the symptoms of AIDS in collaboration with medical and nursing schools, health-care institutions, and professional associations. There is an urgent need for both pre-service and in-service training to increase health-care providers’ awareness of HIV as well as their abilities to provide HIV testing and counseling, identify HIV and AIDS-related complications, and appropriately refer people to HIV care and treatment. PITC within and beyond ANC and more pre-service and in-service medical education about HIV will not only prevent vertical HIV transmission but will reduce extremely late HIV diagnosis and facilitate timely access to free, lifesaving antiretroviral treatment for women and other family members.
